# Study of Molecular Mechanisms Involved in the Pathogenesis of Immune-Mediated Inflammatory Diseases, using Psoriasis As a Model

**Published:** 2009-10

**Authors:** E.S. Piruzian, V.V. Sobolev, R.M. Abdeev, A.D. Zolotarenko, A.A. Nikolaev, M.K. Sarkisova, M.E. Sautin, A.A. Ishkin, An.L. Piruzyan, S.A. Ilyina, I.M. Korsunskaya, O.Y. Rahimova, S.A. Bruskin

**Affiliations:** 1 Vavilov Institute of General Genetics, Russian Academy of Sciences;; 2 Center for Theoretical Problems of Physicochemical Pharmacology, Russian Academy of Sciences;; 3 Moscow Municipal Hospital №24, Department of Health

## Abstract

Psoriasis was used as a model to analyze the pathogenetic pathways of immune-mediated inflammatory diseases, and the results of bioinformatic, molecular-genetic and proteomic studies are provided. Cell mechanisms, common for the pathogenesis of psoriasis, as well as Crohn's disease, are identified. New approaches for immune-mediated diseases are discussed.

## Introduction:

Psoriasis (Psoriasis vulgaris, OMMIM 177900) is a chronic inflammatory, recurring, immune-mediated skin disease that involves several other organs and systems. Psoriasis is a complicated genetically based pathology, which involves several groups of genes [1].

The most common clinical manifestations of psoriasis are the appearance of dry, red patches of skin covered with silvery scales. The affected skin is characterized by an increase of the skin cell number and consequent inflammation due to the abnormal keratinocyte differentiation and infiltration of antigen-presenting cells, activation of T-helper cells, and release of proinflammatory cytokines [1, 2]. The appearance of "unaffected" or "uninvolved" skin is normal. However, the gene expression profiling shows that major changes happen in both damaged and undamaged skin of a psoriatic patient, as compared with the skin of a healthy individual [3].

It is supposed that the multi-gene nature of this disease is associated with the presence of several locuses, related to the susceptibility to the disease, known as PSORS1-PSORS9 (Psoriasis Susceptibility) and located on at least 9 chromosomes. Within these chromosomal regions several genes are mapped candidates to be involved in this pathological process [4-6]. In addition, the development of psoriasis may be influenced by several other genomic locuses [7-9]. According to the data appeared in 2008, candidate genes involved in the development of psoriasis may be located on 10 different locuses (PSORS1-PSORS10) [10]. A strong genetic basis of this disease has been confirmed by family and twin studies that show a high percentage of inheritance (up to 80%), as well as higher concordance (about 70%) in monozygotic twins than in dizygotic twins (up to 30%) [8]. However, just like any other multifactorial diseases, psoriasis is influenced not only by genetic factors, but also by the environment. Many environmental factors may play a role in the development of this disease in susceptible individuals: a mechanical damage skin that may lead to the Kebner's effect (development of psoriatic lesions on the site of epidermal trauma), surgical intervention, UV-radiation, high body mass index, excessive alcohol consumption and smoking, stress factors, including physiological, psycho-emotional and cold stressors[6].

It is well known that bacterial, fungal and virus infections can influence the development of psoriasis [11, 12]. Drugs, such as β-blockers [13], angiotensin-converting enzyme inhibitors [14], antimalarial drugs [15], and lithium [16] can also trigger the development of the disease. Studies show that psycho-emotional stresses can provoke psoriasis development in 23% of the patients, medication - in 16%, physical trauma - in 43%, and infections (over all) - in 14% [17]. The other 4%, perhaps, include other triggering agents.

Therefore, the main reason behind the appearance of psoriasis is unknown; however, it is obvious that this disease is a result of the combined influence of several genetic and environmental factors, as well as the patient's lifestyle. Gene profiling studies show that psoriasis is an immune-mediated inflammatory disease, where epidermal cell structure disbalance , as well as abnormal cell growth and differentiation, is the result of molecular stress signals that initiate an improper immune response [3].

A study of the molecular mechanisms of the development of psoriasis was performed on the clinical, bioinformatical, molecular-genetic, and proteomic levels. The ethnic backgrounds of psoriasis patients were taken into consideration, and comparison with the development of other diseases (specifically Crohn's disease and atherosclerosis) was performed. 

## Materials and Methods

Skin samples collection from psoriatic patients was done under local anesthetic with the use of dermatological biopsy punch (4mm). Patients received no systematic PUVA/UV therapy for a month before the skin biopsy (Table 1). Biopsy samples from unaffected skin regions were taken at a distance of 3 cm from the damaged skin region [3, 18, 19]. The study was approved by the local ethic committee at the Genetics Institute of the Russian Academy of Sciences (RAS) and was conducted in agreement with the principles of the Declaration of Helsinki.

**Table 1:** Patient's information.

	Gender	Age	Psoriasis type	PASI	PsA	Inheritance
1	M	22	P. vulgaris	4.0	No	Denies
2	M	36	P. vulgaris	7.2	No	Father has psoriasis
3	M	23	P. vulgaris	1.8	No	Father and brother have psoriasis
4	M	51	P. vulgaris	9.4	No	Mother has psoriasis
5	M	38	P. vulgaris	2.1	No	Denies
6	M	57	P. vulgaris	4.2	No	Father has psoriasis
7	M	51	P. vulgaris	2.1	No	Denies
8	M	40	P. vulgaris	2.8	No	Father has psoriasis
9	M	34	P. vulgaris	3.2	No	Mother has psoriasis
10	M	49	P. vulgaris	11.4	No	Denies

To grade the severity of psoriasis, a Psoriasis Area and Severity Index (PASI) were used. To calculate overall PASI, local PASI for different body parts were first obtained, using the following formula: PASI = fraction x area x (redness x peeling x thickness). Over all, PASI is equal to the sum of local PASI and can differ in range from 0 to 72. Maximum PASI in this study group was 11.4; minimal, 1.8, meaning that all the patients had an acute form of the disease. The presence or absence of psoriatic arthritis accompanying psoriasis is show in column PsA (Table 1).

Collection of atherosclerosis' autopsies was done from postmortem material. To achieve that, the abdominal aorta with signs of atherosclerosis was taken and samples from the tunica intima layer of atheromatous plaques were collected. Autopsies from the healthy part of the aorta were collected in the same manner. Samples were collected from patients who were treated for different diseases (Table 2).

**Table 2:** Pathological diagnosis of patients with atherosclerosis

Patient	Age	Gender	Diagnosis
1	80	F	Encephalopathy. Brain atrophy, internal hydrocephalis. Arthrosclerosis of brain arteries. Type II Diabetes. Sclerosis, pancreatic lipomatosis.
2	70	F	Ischemic infraction of right-frontal lobe. Arthrosclerosis of the brain vessels with stenosis up to 50%. Type II Diabetes. Hypertension. Arthrosclerosis of heart blood vessels with stenosis up to 75%.
3	77	M	Infraction of the brain in the area of the middle brain artery. Type II Diabetes. Hypertension III degree. Ischemic hear disease: atherosclerotic cardiosclerosis.
4	67	F	Chronic stomach ulcers. Chronic intestinal ulcers. Diffuse cardiosclerosis. Postmyocardial infraction cardiosclerosis. Arthrosclerosis of the coronary blood vessels with stenosis of 70%. Type II Diabetes. Arthrosclerosis of brain vessels with stenosis of 30%.
5	75	F	Breast cancer. Phlebothrombosis in the right crus. Pulmonary thromboembolism of lung artery. Encephalopathy. Arthrosclerosis of brain vessels with stenosis of 25%. Chronic obstructive bronchitis, diffuse pneumosclerosis, obstructive pulmonary emphysema. Gastritis.
6	80	M	Large postmyocardial infraction cardiosclerosis. Arthrosclerosis of the coronary blood vessels with stenosis of 75%. Secondary hypertensison. Bilateral nephrosclerosis. Chronic renal insufficiency.
7	64	M	Chronic alcoholic intoxication. Bilateral bronchial pneumonia. Ischemic heart disease: arthrosclerosis of the coronary blood vessels with stenosis of 40%.
8	87	M	Large postmyocardial infraction cardiosclerosis. Arthrosclerosis of the coronary blood vessels with stenosis of 50%. Hypertension of the small blood-circulation circle. Arthrosclerosis of the brain vessels with stenosis of 30-50%.

RNA extraction from biopsies was done by the Qiagen Kit protocol. RNA was cleaned from DNA contamination using DNAse Qiagen®.

Reverse transcription was done using Promega's reverse transcriptase M-MLV protocol.

Real-time PCR was done using the fluorescent-labelled oligonucleotide probes. Reaction was done using the chemical supplies manufactured by the company Eurogene. All of the primers and probes were synthesized by the DNA-Syntez company. GAPDH was taken as a control for the target genes expression. Results analysis was done using PCR reactions with the following conditions: reaction efficiency more than 95%, correlation coefficient no less then 0.99, and slope -3.4 ± 0.2. Method 2-??ct was used to analyze the results of polymerase chain reaction, according to [20].

To study the proteomic profiles, samples of damaged and visually undamaged skin were used. Proteins were extracted and studied using two-dimensional electrophoresis. A silver dye was used to visualize the gel lines. The images obtained were analyzed using the Melanie II program (GeneBio, Switzerland). Protein identification was done using the methods of MALDI-TOF mass-spectrometry and nano LC-MS/MS mass-spectrometry at the Proteomic center of the Institute of Biomedical Chemistry of the Russian Academy of Medical Sciences (RAMS). 

The GEO DataSets (http://www.ncbi.nlm.nih.gov/geo/) database was used to for bioinformatics analysis. The GEO DataSets database contains a number of electronic tables where the gene expression microarray results are collected. The MetaCore® program produced by GeneGo Inc (USA) was used for the analysis. The MetaCore® program established that the lowest p-value correlates to the higher possibility of gene involvement into the process. The original threshold for the p-value was established as equal to 0.05. 

## Results and Discussion:

### The Role of Ethnic Background in the Development of Psoriasis 

During the last several decades, extensive knowledge has been collected showing that in addition to individual differences in the metabolism of medical compounds and system reactions to those compounds, as related to their therapeutic and side effects, there are also individual differences in people's susceptibility to diseases. The investigation of the factors that influence an individuals susceptibility to a specific disease and a patient's response to treatment was started in the 1970s under the leadership of L.A. Piruzyan [21, 22]. The necessity of tracking the kinetics of metabolic changes during the process of chemical compound interaction with biological objects was stressed [23].

**Fig. 1.**
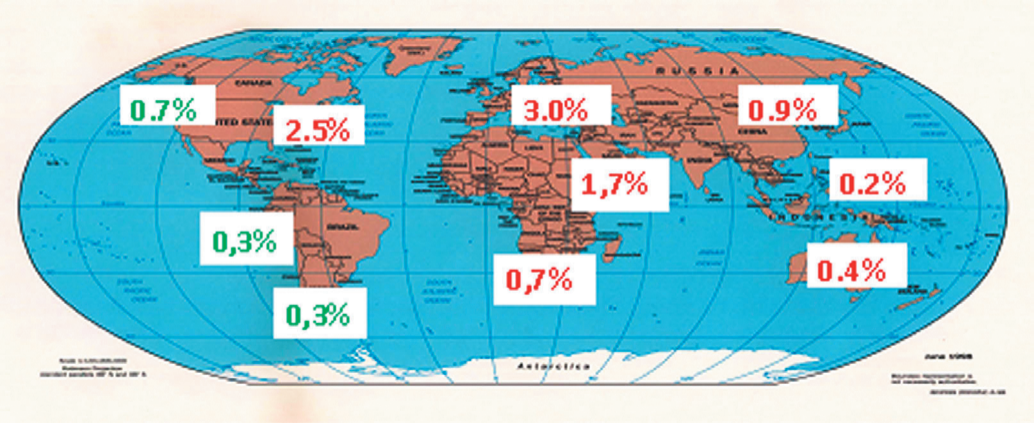
Frequency distribution of psoriasis case rates in different regions of the world. The percentage of case rates in Aborigines is marked in green

One's ethnic background also plays a very important role in the development of complicated diseases, such as psoriasis. On average, 2-3% of the world's population suffers from psoriasis. However, these data vary considerably depending on the country, geographic region, and ethnic background of the patient [24]. There is a huge geographic variation in the occurrence of psoriasis (Figure 1) [25]. The huge difference in the diseases incidence between Asia and Europe is proof of the significance of the ethnic component in the development of psoriasis. It is obvious that a regional-ethnic differentiation in the occurrence of psoriasis exists [7, 8, 26]. However, data on the occurrence of psoriasis collected in different populations is somewhat contradictory, which could mean that different analytical methods were used in the collection of the data. In order to make data collection easier and to standardize the analytical methods, we developed an Individual Information Card (IIC), which includes more than 50 grading profiles [27, 28]. The analysis of the genetic structures of samples from Dagestan by the IIC method, as well as family pedigrees, showed high ethnic purity, which is a result of very few marriages across ethnic groups. The consequence of this is a high level of endogamy, which leads to a rise in the degree of homozygosis in the population. Besides, the study of the genealogical trees includes a large number of families with multiple children, which provides an easier way of tracking families' structures in the space of 3-4 generations. For example, the average number of children in the families of Avarecs, Dagestanis, Lezgins, Lakcevs, Azerbaijanis, and Nogaicis is 3; the average number of children in the families of Kalmyks, Tabasarans, and Agul's is 4; but in Russian families the average number of children is only 1. It should also be noted that in such families several generations live fairly close to each other and, therefore, are influenced by similar environmental factors.

Our study showed a significant difference in the presence of complications and duration of the disease depending on the type of illness (Type I - early, Type II - late) in the different ethnic groups of Dagestan. The data obtained in this study show the important role of the ethnic component in the inheritance of such a complicated pathology as psoriasis. The data also show that it is possible to use IIC and a model population from Dagestan to study the type of psoriasis inheritance with reference to the ethnic component [29].

A psoriasis sensitivity gene study done on samples obtained from Russian, Tatarstan, Bashkortostan, and Hakasiyan ethnic groups in the republics of Bashkortostan and Hakasiya showed that the polymorphic gene locuses HLA-C and HCR are the basic sensitivity markers for psoriasis in the analyzed regions, regardless of the form of the disease and the ethnic group [30].

In order to study the incidence of psoriasis and for gene expression profiling, it is necessary to align all the data according to all the parameters of the study. Several differences in gene expression using Affimetrix-chips in Japanese patients with psoriasis and the results obtained with a similar method in four other study groups show variability in gene expression in individual patients based on age, gender, and environmental factors [3, 31, 32, 33, 34].

### Analysis of Biological Microarrays

**Fig. 2.**
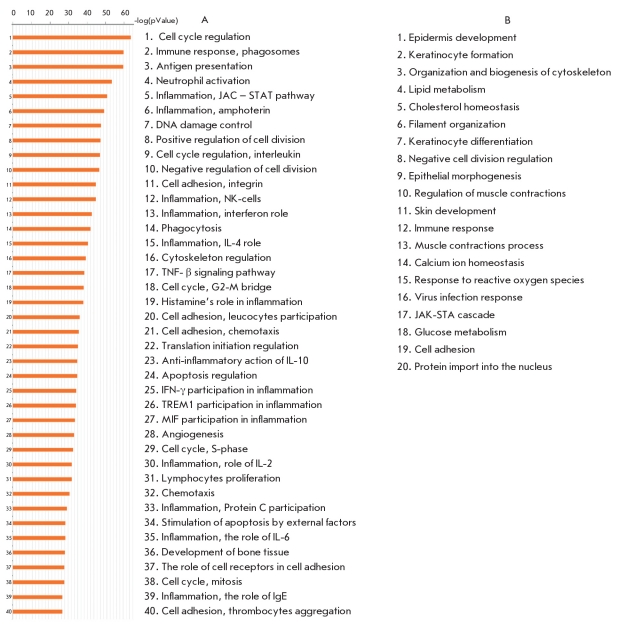
Forty most altered processes (from the most altered to the less altered) in psoriatic skin compared to the skin of healthy volunteers (A) and twenty most altered processes (from the most altered to the less altered) in psoriatic lesions compared to the unaffected skin (B)

Today, the use of methods that allow to systematize and compress a vast pool of genetic information is widespread. This allows to explain different gene interactions. One such method is MetaCore® a program built by GeneGo Inc. (USA). As a result of gene expression microarray analysis of at least 12,000 genes using MetaCore® we concluded that 7,563 genes changed expression at least 1.5 times [35]. Figure 2 contains the list of processes changed in psoriasis-. The main processes that change under the influence of psoriasis are the immune response, cell cycle, inflammatory response, proliferation, and others. The process of disease development involves a number of immune system cells, such as T-lymphocytes (Th1, Th17), antigen-presenting cells (APC) dendrite cells, Langerhans cells, macrophages., and natural killer (NK) cells [36]. Cell-to-cell signaling is done with the help of signaling molecules - cytokines, as well as by the interactions of various cell receptors with their ligands. For example, receptors TLR (Toll-like receptors) belong to the family of receptors that promote an immune response when a viral or microbial presence is detected. These receptors are expressed not only on the surface of immune system cells (monocytes, macrophages, dendrite cells, and granulocytes), but also on the surface of the epithelial cells in the respiratory tract and the skin - important regions of host - pathogen interaction [37]. The TLR family includes 11 receptors, and its ligands include lipopolysaccharides, single-stranded or double-stranded RNA, lipoproteins, flagellin, and unmethylated DNA [38]. Stimulation of these receptors results in stimulation of an entire complex of signaling cascades, including NF-kappaB/Rel, IRF and MAPK - ERK, JNK, and p38. For example, MAPK induces the expression of AP-1 family transcription factors, such as Fos and Jun, and activates transcription activity in the protein complex AP-1, starting several processes leading to inflammation [39]. As a result, the expression of anti-inflammatory cytokines (IL-6, IFN- γ, IL-12 and TNF- γ) in both induced and co-stimulating molecules, such as CD80 and CD86, activates the immune response [40, 41]. Considering that viruses and bacteria are the main triggers of psoriasis, these receptors play an important role in the initiation of the disease [38, 42].

In the entire family of chemokines, a high level of expression was noticed in CCR4, CCR5, CCR6, CCR10, and CXCR3. Chemokines belong to the group of anti-inflammatory cytokines and provoke an influx of Th1 cells into the inflamed regions [43]. Expression of receptors CCR4 and CCR10 usually occurs in CD4+ and CD8+ memory T-cells with CLA + phenotype (cutaneous lymphocyte antigen), which decides on the lymphocyte migration into the lymphatic nodes [44]. A ligand for the receptor CCR4 is CCL17, which is expressed on the endothelial surface of dermal blood vessels, along with ligand CCL27, and provokes the migration of leucocytes into the skin [45]. An expression level of ligand CCL27, which is a ligand for receptor CCR10, occurs in keratinocytes under the influence of anti-inflammatory cytokines IL-1 and TNF-α. In this way, interaction of CCR10-CCL27 involves T-cells in the inflammatory process in the skin [46]. CXCL16 in atherosclerosis can be used as an example of the regulation mechanism of chemokine expression. Expression of this chemokine is induced via IL-18 through the following signaling pathway: MyD88→ IrAK1-IrAK4-TRAF6 (tumor necrosis factor receptor - associated factor 6)→ c-Src→ PI3K→ AKT→ JNK→ AP-1 [47].

Data shows that the transcription factor AP-1 acts as a common link of the regulatory pathways of TLR-receptors and chemokines.

### Analysis of Gene-to-Gene Interactions in Psoriasis 

**Fig. 3.**
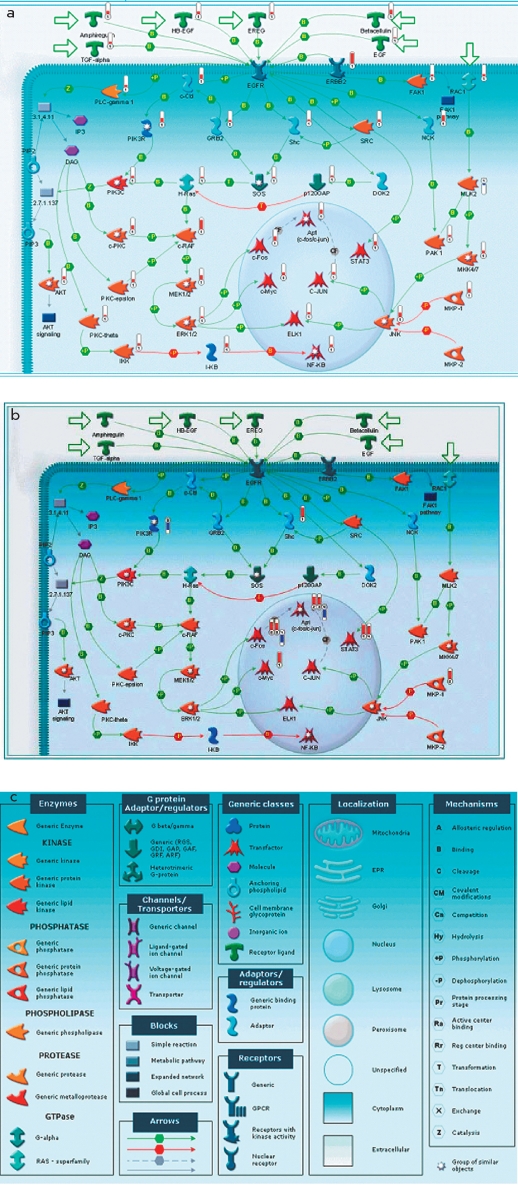
Signal transduction pathway from EGF to AP-1 and alteration of expression of some genes responsible for this process during psoriasis in skin (A - skin of psoriatic patients compared to the skin of healthy volunteers, B - in affected skin compared to the unaffected skin of psoriatic patients, C - the notation conventions). There are also designations used on the maps that characterize the alteration of the level of expression of genes: - downregulation ; upregulation

Signal transmission from EFG (Epidermal Growth Factor) receptor inside the cell, shown in figures 3a and 3b, can be used as an example of gene-to-gene interaction in psoriasis. Both figures show that genes coding transcription factors, such as c-Fos, c-Myc, c-Jun and ELK1, are activated via signal transmission from EGFR through GRB2 and Shc to SOS, and then through the following pathway: h-Ras> c-RAF> MEK1/2> ERK1/2> transcription factors. Regardless of the fact that the expression levels is changed only for some genes coding corresponding mediator signals (GRB2, Shc, SOS, h-Ras, c-RAF, MEK1/2), like, for example c-RAF (Figure 3a), expression of transcription factor c-Fos, c-Myc, c-Jun, ELK1, and STAT3 turned out to be a lot higher in cases of damaged skin than in the undamaged skin of psoriatic patients, and higher than in the skin of healthy volunteers [35].

**Fig. 4.**
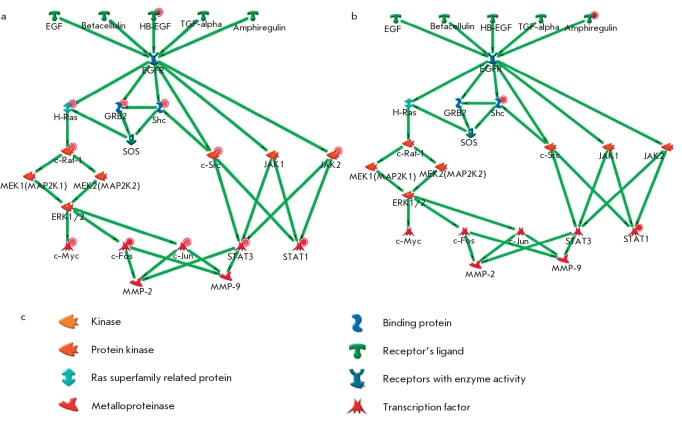
Level of expression alteration of EGF signal transduction pathway genes in the skin of psoriatic patients: A- as compared to the skin of healthy individuals, B- in affected skin compared to the phenotypically normal skin of the same patients, B - the notation conventions. The hue of the color depends on the absolute value of the expression change (the bigger the change, the more saturated the color)

Figure 4 shows the degree of change in the EGF gene signaling pathway expression in the skin of psoriatic patients. We compared the threshold change (Fold Change) of gene expression in psoriasis lesions as compared to the normal skin (Figure 4a) and phenotypically healthy skin of some patients. Fold Change (FC) of expression was established as 2. Figures 4a and 4b show some protein-to-protein interactions that are the part of signaling pathways induced by the Epidermal Growth Factor (EPF) and its ligands. Green arrows show consecutive activation of protein interaction either through bounding or phosphorylation. It is noticeable that the expression of some EGFR-ligands is higher in the psoriasis-damaged skin (HB-EGF and amphiregulin - expression increased 5.77 and 4.96 times, accordingly). Figure 4a shows that each gene coding the proteins of the signaling pathway is upregulated; for example, c-Raf kinase with FC = 4.46 and c-Src with FC = 3.96. Moreover, transcription factors themselves are upregulated as well. Therefore, the expression of c-Myc is increased 6 and 15 times; c-Jun, 3.35 times; STAT1, 17 times; and c-Jun, 34 times. The general picture of gene expression changes is shown in Figure 4b. STAT3 is upregulated 8.6 times. This leads to the conclusion that the cells involved in this pathological process are characterized by a significant change in the levels of gene expression, involving a significant number of various molecular pathways and their associated transcription factors in this complicated process. A close look needs to be taken at the overexpression of those transcription factors, since under normal conditions these transcription factors do not show such sudden changes in the expression levels. The uninvolved - meaning visually healthy - skin of psoriatic patients is somewhere in between the skin of the healthy individual and the inflamed skin of the patient, since the difference between samples collected from the visually healthy skin of a psoriatic patient and damaged skin is significant, but not as significant as the difference between the damaged skin of a patient and the skin of a healthy individual.

After the analysis of gene interactions, we concluded that in all of the processes studied the main transcription factors that changed their expression in psoriasis, only components of transcription factor AP-1 and transcription factor NF-kB are present. Transcription factor NF-kB is activated during the immune response. In this particular study, we analyzed the role of components of transcription factor AP-1 [48].

### Transcription Analysis

Our group had a significant interest in the comparison of the level expression of genes in the damaged skin regions of psoriatic patients as compared to the expression of the same genes in the visually unaffected skin regions located no farther than 3cm from the affected regions. This kind of comparison was done in several other laboratories around the world [3, 18, 19], and it allows for maximum purity of the experiment. A threshold of change of the expression levels was established for each of the genes equal to 2. Based on a search of the literature and several databases, we established a number of genes that may be important for experimental study. These genes include genes that code transcription factor AP-1 (C-JUN, JUNB, JUND, C-FOS, FOSB, FRA-1, FRA-2, and others).

**Fig. 5.**
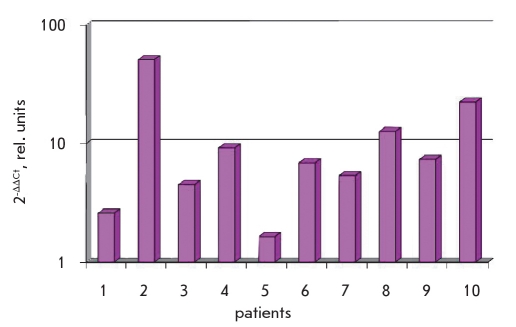
Level of expression alteration of FRA-1 in affected psoriatic skin relative to the visually unaffected skin of the same patients (1- absence of expression alteration; <1 - decrease of expression in affected sample;>1 - increase of expression in affected sample)

Using a real-time polymerase chain reaction, we were able to analyze the levels of gene expression for 12 genes in the damaged skin and compare it with the visually unaffected skin of the same psoriatic patient (data not shown). Results of this experiment showed that practically all patients showed a more than doubled expression of gene FRA-1 in the damaged skin compared to the visually unaffected skin regions (Figure 5). All other genes differed in the direction of change of their expression.

For comparison, we were able to analyze the expression levels of gene FRA-1 in the samples obtained from the atherosclerotic patients. 

**Fig. 6.**
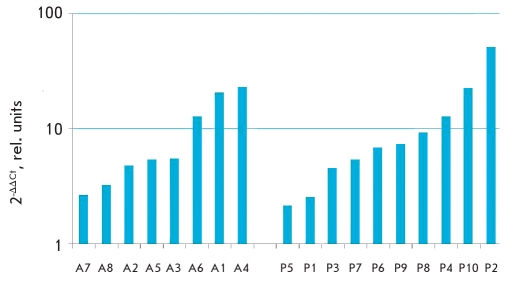
Level of expression alteration of FRA-1 gene in atherosclerosis affected vassels compared to visually unaffected (A1-A8) and psoriasis affected skin compared to unaffected (P1-P10). 1- absence of expression alteration; <1 - decrease of expression in affected sample;>1 - increase of expression in affected sample)

Results obtained with real-time PCR showed an increase in the expression of gene FRA-1 in all patients in the atherosclerosis-affected region of the blood vessels and those suffering from skin psoriasis (Figure 6). At the same time, pathological activation of FRA-1 expression, as a component of AP-1, can lead to an increase in IL-2 expression and the consequential stimulation of auto-reactive cytotoxic T-lymphocytes, followed by an increase in the production of anti-inflammatory cytokines and autoantibodies, resulting in the development of a local inflammatory process, which is common in the appearance of psoriasis and atherosclerotic lesions [49, 50]. Increased expression of FRA-1 can also lead to an increase in the expression of IL-18, which induces transcription and expression of MMP9 (matrix metalloproteinase 9) and stimulates production of the active form MMP9. This stimulation leads to an increase in smooth muscles cell migration, which is an important part of the appearance of atherosclerotic plaques [51]. Therefore, we can conclude that it is possible that FRA-1 plays a crucial role in the pathogenesis of psoriasis and atherosclerosis.

Therefore, with the help of the bioinformatic analysis, the psoriatic gene networks were examined to detect the presence of relatively closed processes, and the main genes were identified: the regulators of the transcription complex AP-1. Transcription profiling of selected genes (C-JUN, JUNB, JUND, C-FOS, FRA-1 and FRA-2) based on RNA of 6 chosen genes showed a multidirectional regulation of these genes in psoriasis (data not shown). Gene FRA-1 stands out from the other genes analyzed that code the proteins of AP-1 complex (shown on Figure 5). Moreover, change in the expression of this gene before and after treatment showed a clear tendency toward a lowering of the levels of mRNA of this gene, which correlated with the positive dynamic in the patients illness history (data not shown). Therefore, the transcription activity of FRA-1 gene complex AP-1 may be a sort of efficiency indicator of treatment at the molecular level.

### Proteomic Study

The final goal of our study was to analyze the changes occurring in the metabolic pathways which lead to the pathogeneses, since on that level we can identify new targets for medical treatment and new approaches to pharmacological therapy can be found.

With the help of the proteomic analysis of psoriatic skin samples, we were able to establish 10 protein markers present only in the psoriasis-affected skin samples but absent in the unaffected skin samples (Table 3) [52]. A few protein families were of the biggest interest.

**Table 3:** Proteins identified as different at involved and uninvolved psoriatic skin [52]

Blot, number	Protein name	%Vol	
		Affected skin	Unaffected skin
1	Keratin 17		
	Keratin 14		
	Keratin 16		
2	SCCA2/SCCA1		
3	Squamous cell carcinoma antigen; SCC antigen		
4	Enolase 1		
5	Superoxide dismutase [Mn]		
6	Galectin 7; Gal - 7		
7	Protein S100 - A9		
8	Protein S 100 - A9		
9	Protein S100-A7 (Psoriasin)		
10	Protein S100-A7 (Psoriasin)		

The S100 family includes at least 13 proteins, and the genes that code them are localized in cluster form on the 1st chromosome (1q21), which correlates to the psoriasis susceptibility locuse PSORS4. It is known that this protein is highly expressed in the psoriasis-affected skin; however, today this gene is no longer considered a candidate for the development of psoriatic processes. At this point, the exact role of this protein remains unknown.

Proteins SCCA1 and SCCA2 belong to the family of serine proteinase inhibitors. Some studies [53] have shown that protein SCCA1 is expressed in the normal skin, while in the psoriasis affected skin its expression is twice higher.

Protein SCCA2 is not expressed in the control samples, and in the psoriasis-affected skin samples its expression levels are similar to that of SCCA1. 

These proteins can be considered as potential targets for the pharmacological compounds in psoriasis treatment [54].

### Comparative Analysis of Psoriasis and Crohn's Disease Pathogenesis

**Fig. 7.**
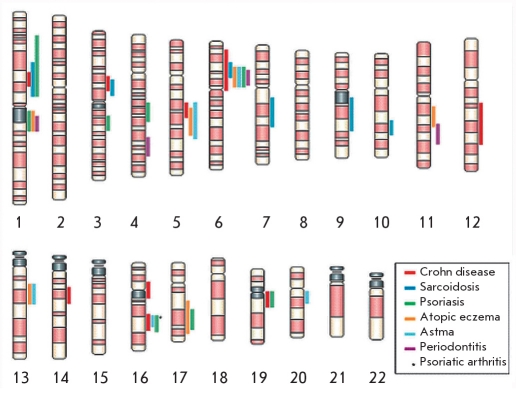
Clustering of linkage regions and disease genes in human inflammatory barrier disease [55]

A multidimensional study of the molecular mechanisms of psoriatic pathogenesis can be considered as a model for the study of the pathogenesis of other immune-mediated inflammatory diseases (IMID - Immune-mediated inflammatory disorders), which are characterized by acute and chronic inflammatory conditions. The most common IMID-phenotypes are Crohn's disease, ankylosing spondylitis, rheumatoid arthritis, psoriasis, uveitis, and psoriatic arthritis. The central role in all of these diseases is played by the cytotoxin TNF-? (tumor necrosis factor - alpha). Figure 7 shows the localization of gene groups responsible for the development of these diseases [55]. Crohn's disease and ulcerative colitis are chronic inflammatory diseases that are generally called IBD (inflammatory bowel disease). Both twin studies and the generally higher incidence of these diseases in close relatives point to a genetic basis for these diseases. The increase in the number of cases in Europe and North America since the second half of the 20th century is an indication of the strong influence of environmental factors, with relatively low levels of concordance in the monozygotic twins (~50% for the Crohn's disease and 10% for the ulcerative colitis). Even though animal studies do provide clues about the etiology of Crohn's disease, its nature remains a mystery [55, 56].

**Fig. 8.**
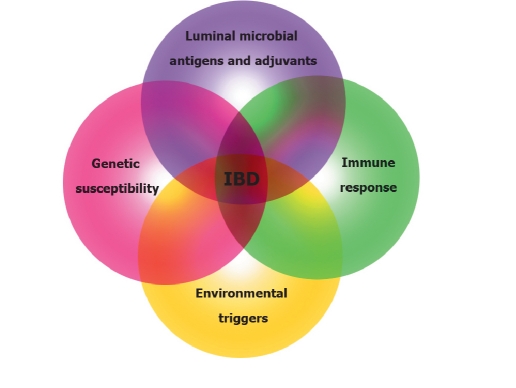
The interaction of different factors (genetic susceptibility, environmental triggers, luminal microbial antigens and adjuvants, immune response) that cause chronic-inflammation intestine process[56]

The most whidespread hypothesis about IBD pathogenesis is the presence of a heavy T-cell immune response to certain environmental factors and some pathogenic enterobacteria in the genetically susceptible individuals, which increases the beginning or reactivation of the disease. Figure 8 shows the activation of four independent components, the interaction of which at different points of the diseases development is necessary for its clinical implications. From our point of view, comparable studies of the genetic factors involved in the pathogenic pathways of psoriasis and Crohn's disease can play a significant role in the deciphering of the molecular mechanisms of the pathogenesis of these diseases. A comparative bioinformatics analysis of microarray database GEO Data Sets allowed to prepare a general list of the genes that undergo changes in the cell processes of both diseases.

### Identification of genes that change their expression in both pathologies - psoriasis and Crohn's disease

Our research was focused on the identification of differentially expressed genes in each data set and comparison of these lists of genes at the systemic level. The approach we took in working on this problem was dictated by the character of the expression data (high noise level, large amount of data to be analyzed) and the attributes of each individual data set taken for the analysis (they were done on different types of microarrays that have different numbers of probes and, therefore, cannot be compared directly) [57].

Originally, psoriasis data sets contained information on the expression of 12,626 probes for 8 experiments (4 samples of damaged skin and 4 samples of healthy skin). When probes with badly detectable expression were thrown out, the number of probes was reduced to 5,076. The list of probes with statistically significant differences in expression between the affected and unaffected skin samples contained 410 probes with a significance level of 0.1.

The data sets for Crohn's disease contained information on expression levels for 24,016 probes in 21 experiments (10 intestinal epithelial samples of healthy individuals and 11 epithelial samples of damaged epethilia). The list of probes with statistically significant differences in expression between the damaged and undamaged tissues was 3,850 probes, with a significance level of 0.1. Such a huge difference in the probe lists sizes was due to the fact that the algorithm to control Type I errors (FDR) depends on the size of the input set: the larger the number of genes in the input set, the larger the number of genes taken with the same p-value distribution that will go through FDR control. In our study, the number of analyzed probes in the Crohn's disease data set is five times that of the psoriasis data.

The resulting data sets of differentially expressed genes were introduced into the MetaCore® Since microarrays contain not only probes for the genes studied, but also for a large number of EST with unclear roles, in this step the size of the genes lists changed because not all of the probes have a correlating gene in the MetaCore® database, and because some probes correlate to more than one gene. The size of the genes lists changed to 425 genes for psoriasis and 2,033 for Crohn's disease. 

These genes lists had 49 common genes, and the level of similarity is rather significant (p-value = 4.94x10-2). Fisher's test was used to grade this significance; 9,017 genes present in the two study data sets were taken. Identification of these genes was done by comparing the genes lists for both gene microarray sets in MetaCore®.

Forty-nine genes were selected for the further analysis and are shown in table 4. The cell molecular-genetic processes associated with the genes common to psoriasis and Crohn's disease were of a particular interest. Program methods in MetaCore® were used to create Table 5, where the most possible cell processes involving genes from Table 4 are shown. The processes shown in Table 5 can be divided into two categories: those participating in inflammation processes and those participating in the regulation of the cell cycle. In both psoriasis and Crohn's disease, the main pathological focus lies in the focus of inflammation. Cell cycle and apoptosis are disturbed in psoriasis. Keratinocytes in the focus of the inflammation have no time to apoptose due to extra proliferation; thus, they form the skin patches we observe in psoriasis. Similar processes take place in the intestine of Crohn's disease's patients.

**Table 4:** The list of genes common for pathogenesis of psoriasis and Crohn's disease.

Gene	Identification Number	Location	Gene	Identification Number	Location
GNA15	2769	19p13.3	CBX3	11335	7p15.2
GPM6B	2824	Xp22.2	UGT1A6	54578	2q37
IFI44	10561	1p31.1	DEGS1	8560	1q42.11
OAS2	4939	12q24.2	PSME2	5721	14q11.2
FOXC1	2296	6p25	TRAK2	66008	2q33
ZNF207	7756	17q11.2	DNAJC7	7266	17q11.2
IFI35	3430	17q21	CSNK1D	1453	17q25
STAT3	6774	17q21.31	TRIM22	10346	11p15
TXNDC1	81542	14q22.1	IRF9	10379	14q11.2
MRPL9	65005	1q21	UBE2L6	9246	11q12
CASP4	837	11q22.2-q22.3	ETS2	2114	21q22.2
MECP2	4204	Xq28	QPCT	25797	2p22.2
LONRF1	91694	8p23.1	SFPQ	6421	1p34.3
CG018	90634	13q12-q13	UGT1A4	54657	2q37
VKORC1	79001	16p11.2	H2AFY	9555	5q31.3-q32
MIB1	57534	18q11.2	HMGN1	3150	21q22.2
RFK	55312	9q21.13	CTSC	1075	11q14.1-q14.3
SOSTDC1	25928	7p21.1	SERPINB5	5268	18q21.3
KIAA1033	23325	12q24.11	IER2	9592	19p13.13
SYNCRIP	10492	6q14-q15	S100A8	6279	1q21
RARG	5916	12q13	ARMET	7873	3p21.1
DDOST	1650	1p36.1	FGFR2	2263	10q26
CDC42EP1	11135	22q13.1	RBPMS	11030	8p12-p11
S100A9	6280	1q21	JUNB	3726	19p13.2
PHGDH	26227	1p12			

**Table 5:** The common cell processes typical for psoriasis and Crohn's disease

Processes	P-value
Inflammation: interferon signaling pathway	
Signal transmission: WNT signaling pathway	
Regulation of translation initiation	
Morphogenesis of blood vessels	
DNA repair	
Inflammation: amphoterine signaling pathway	
Cell cycle and apoptosis driven proteolysis	
Interleukin regulation of cell cycle in G1-S phase	
Signal transmission: androgen receptor signaling pathways	

### Reasonable approaches to the pharmacological treatment of psoriasis and possible new approaches to the treatment of immune-mediated inflammatory illnesses

Psoriasis is considered a recurrent incurable illness; its treatment focuses on lengthening the remission periods and reducing the severity of the disease. The drugs used (cyclosporine, system retinoids and fumarates) lead only to a temporary improvement of the patient's condition. Nonetheless, some new approaches are being explored. New therapeutic methods are characterized by a more specific influence on the specific molecular targets that play a key role in the formation of the pathological processes in psoriasis. They include modificators of the biological response, such as alefacept, efalizumab, etarnecept, infliksimab, and adalimumab, which specifically target the molecular mediators involved in the immune-pathogenesis of psoriasis (receptors and ligands). For example, the suppression of TNF? activity, a key cytokine in the innate immune response, is achieved through three inhibitors of TNF? (alefacept, etarnecept, and infliksimab). Etarnecept is a two-component protein produced from the ligand-binding fragment receptor TNF attached to the Fc-fragment IgG1. It binds TNF and blocks its interaction with cellsurface receptors, reducing the inflammation process. Infliksimab - TNFα human monoclonal antibodies lower the activity of TNF?, which lowers the production of IL-1 and IL-6 [58]. Therefore, the new generation of biological approaches to the treatment of psoriasis focus on the specific destruction of targets for the T-cell mediated pathogenic processes.

A number of successful attempts at psoriatic arthritis treatment with bio-modificators have been documented since 2001 [58, Abstract book of Third EAD International Spring Symposium, Sofia, 2005. TNFα levels are increased in the intestinal mucosa during Crohn's disease; therefore, TNF? inhibitors are used to treat this disease. This kind of monotherapy by means of bio-modificators is used for the treatment of psoriatic arthritis and rheumatoid arthritis [59, 60]. However, a number of unusual side effects associated with this treatment have been reported. For example, some reseachers have indicated the development of psoriatic lesions on the skin of some Crohn's disease patients [60, 61, 62]. The mechanisms leading to such serious side effects are unclear; therefore, continued research in this area on the molecular's genetic level is necessary.

In conclusion, the main goal of investigations of the pathways leading to the pathogenesis of immune's ediated diseases on the molecular level is to find new pharmacological treatment options. The proteomic studies of skin samples affected by psoriasis and intestinal fragments affected by Crohn's disease performed by our research group are geared toward finding new targets for pharmacological treatment options. It is also necessary to compare the obtained data with the results of the meta-analysis of development pathways for these two immune-mediated inflammatory diseases done by us.

We used psoriasis as an example to show the necessity of an integrated approach to the deciphering of the disease's pathogenesis stages, including clinical and bioinformatical analysis, as well as analysis of metabolic and genetic data, which will assist in the development of simple methods of individual characterization of pathogenesis and finding more effective methods for the treatment of each individual. In this manner, psoriasis is taken as a typical complicated disorder and is considered an adequate model for the study of the pathogenicity mechanisms for other disorders similar to psoriasis in their influence on human life. 
